# Uptake of postnatal care and its determinants in Ethiopia: a positive deviance approach

**DOI:** 10.1186/s12884-022-04933-3

**Published:** 2022-07-27

**Authors:** Samrawit Mihret Fetene, Tsegaye Gebremedhin

**Affiliations:** Department of Health Systems and Policy, Institute of Public Health, College of Medicine and Health Sciences, University of Gondar, P. O. Box: 196, Gondar, Ethiopia

**Keywords:** Postnatal care, Uptake, Positive deviance, Women with no education, Multilevel-mixed effect, Ethiopia

## Abstract

**Background:**

Postnatal care (PNC) services are an essential intervention for improving maternal and child health. In Ethiopia, PNC service has been poorly implemented, despite the governments and partners’ attempt to improve maternal and child health service utilization. Moreover, many literatures identified that women with no education are significantly underutilized the PNC services. Thus, this study aimed to assess the PNC service uptake among women at high risk for underutilization of PNC services and to identify the individual and community level determinants of PNC services uptake in Ethiopia using the positive deviance approach.

**Methods:**

Data from the Ethiopia Demographic and Health Survey 2016 were used. A total of 2417 deviant women (women with no education) were identified through a two-stage stratified sampling technique and included in this analysis. A multilevel mixed-effect binary logistic regression analysis was computed to identify the individual and community-level determinants of PNC services uptake among deviant women. In the final model, a *p*-value of less than 0.05 and adjusted odds ratio (AOR) with 95% confidence interval (CI) were used to declare statistically significant determinants of PNC services uptake.

**Results:**

In this analysis, the uptake of PNC service among deviant women was 5.8% [95% CI: 4.9–6.8]. Working in the agriculture (AOR = 2.15, 95% CI: 1.13–3.52), being Orthodox religion follower (AOR = 2.56, 95% CI: 1.42–4.57), living in the highest wealth quantile (AOR = 2.22, 95% CI: 1.25–3.91) were the individual level determinants, whereas residing in the city administration (AOR: 3.17, 95% CI: 1.15–8.71), and living closer to health facility (AOR: 1.57, 95% CI: 1.03–2.39) were the community level determinants.

**Conclusion:**

The study highlighted a better PNC service uptake among deviant women who are working in the agriculture, follows orthodox religion, lives in highest household wealth status, resides in city administration, and living closer to the health facility. The positive deviance approach provides evidences for health policy makers and program implementers to improve health behavior in specific target population, and ultimately to bring better maternal and child health outcomes, despite acknowledged adverse risk profile. Such strategy and knowledge could facilitate targeted efforts aimed at achieving national goals of maternal and newborn mortality reduction in the country.

## Background

The interval between birth and 42 days after giving birth is a postnatal period where the majority of maternal and infant deaths occur [1]. The mother’s life is most at risk immediately after birth from bleeding and infection, while premature birth, hypoxia, and severe infections pose the greatest risk to newborns [1, 2]. But, majority of the maternal and neonatal deaths can be averted through a better intrapartum and early postpartum health care services for the mothers and newborns [3].

In 2020, the World Health Organization (WHO) report showed that the global maternal mortality ratio was 152 deaths per 100,000 live births [4]. The report also estimates that more than 5 million children died before their fifth birthday [5]; of these 2.4 million newborns died in 2020 [6]. Unfortunately, low- and middle-income countries account for 99% of maternal and neonatal deaths [7].

In Ethiopia, a substantial proportion of women are dying due to direct and indirect obstetric causes [8]. According to the Ethiopia Demographic and Health Survey (EDHS) report, Ethiopia had a maternal mortality ratio of 412 maternal deaths per 100,000 live births in 2016 [9]. To alleviate this problem, the Ethiopian government and other partners have been implementing the postnatal care services through expanding the primary health care including user free services, home based postnatal care by the health extension workers [9, 10]. Providing postnatal care services (PNC) for the mother and her baby is one of the recommended strategies for reducing maternal and neonatal complications during the postpartum period [11]. But, despite the government’s focus to improve the maternal and newborn health trough a better intra- and post-partum care, the uptake of PNC is still very low and have been implemented poorly in Ethiopia [12]. As a result, only 17% of women and 13% of newborns were received PNC services within 48 hours after birth in 2016 [9].

Previous empirical studies were identified the factors influencing the PNC services uptakes by focusing on all women of reproductive age. The socio-demographic and economic variables like mothers’ educational level, residence, household wealth index, marital status, religion, age and occupations [13–21], previous obstetric characteristics like having antenatal care (ANC) visit, place of delivery, wanted pregnancy, parity, age at first birth, [13, 14, 16, 17, 20, 22–24], and also health services related factors like distance from health facility [23, 25] were the identified factors.

However, studies using the positive deviance (PD) approaches to explore factors influencing the uptake of PNC services are still limited, as most existing literatures focuses in general on all women of reproductive age [10, 15, 16, 21, 22, 26, 27]. Identifying mothers with positive health behaviors despite an adverse risk profile and exploring their characteristics could help health policy implementation, enable program managers to optimize maternal and child health initiative’s performance, and ultimately improve health outcomes. Thereby, knowing the positive influencer of PNC uptake among women with the high risk for services underutilization can be used to orient high impact interventions to improve the health services utilization in resource constrain settings; Ethiopia. Moreover, since majority of the women in Ethiopia are not going to formal education and living in the rural community, identifying the positive determinants of PNC uptake among these women might be essential for designing better strategies to improve the health care delivery subsequently to meet the sustainable development goal 3.1 and 3.2. Therefore, the study was aimed to identify the PNC service uptake among women at high risk for underutilization of PNC services and to explore factors that characterize the PD women for PNC services uptake in Ethiopia.

## Methods

### Data sources and context

The study used the EDHS 2016 data, a nationally representative household survey that has been implemented by the Central Statistical Agency (CSA) of Ethiopia every 5 years [28]. Ethiopia is a home country of an estimated 114 million population (CSA 2015). Administratively, the country is divided into nine regions (Tigray, Afar, Amhara, Oromia, Benishangul, Gambela, South Nation Nationalities and Peoples’ Region (SNNPR), Harari and Somali) and two City Administrations (Addis Ababa and Dire-Dawa). Those nine regions can be divided in to developed regions (Tigray, Amhara, Oromia, South Nation Nationalities and Peoples’ Region (SNNPR), and Harari), and emerging regions (Afar, Benishangul, Gambela, and Somali).

### Sampling procedures and sample size

The 2016 EDHS used the 2007 Ethiopian population and housing census as a sampling frame, which was conducted by the CSA of Ethiopia and a complete list of 84,915 enumeration areas (EAs) were used in the census. The 2016 EDHS sample was stratified in two stages. A sample of EAs were selected from each stratum, independently. Then a total of 645 EAs were selected with probability proportional to the EA size, and each sampling stratum was selected from the given samples. The total residential households in the EA were the EA size, and a household listing operation was implemented. Then, the resulting lists of households were used as the sampling frame for selecting households in the second stage. Accordingly, all women aged 15–49 years who are regular members of the selected households were eligible for the survey. Finally, from a total of 4081 women identified from the EDHS 2016, 2417 deviant women were included in this analysis, and data were extracted from the datasets using STATA version 14 software. Variables at the individual and community-level were also extracted and further analyzed.

### Identification of the positive deviant women

We used the Anderson’s behavioral model of health service use [29] and other related studies [30, 31] to identify the positive deviant for PNC services uptake. Accordingly, education is the major determinant of health services utilization. We selected women who had no education as a sub-group with a very low likelihood of PNC services utilization, as education was the strongest predictor of PNC after adjusting for the other risk factors associated with PNC in this population. Positive deviant women were those who had no education but had an adequate uptake of PNC services. Finally, in the analysis, we compared the characteristics of the PD women to those of their counterparts. Due to significant variations by clusters in the overall use of PNC and also the individual and household level data were nested under the community level data, the analysis was stratified by individual and community level.

### Measurements of variables

Uptake of PNC services among deviant women was the dependent variable. The uptake was assessed when a woman received PNC services within 2 months after delivery, irrespective of their place of delivery. The information on the uptake of PNC services for their recent birth was assessed based on the women’s verbal responses during the survey. Accordingly, it was categorized as “yes” if a woman received at least one PNC visit, otherwise “no”.

Individual-level variables; socio-demographic and economic variables (age, occupational status, religion, marital status, age at first birth, desire for child, household wealth status) were included in this analysis. On the other hand, place of residence, region, living closer to health facility, and media exposure were the community-level variables. Household wealth status was assessed using the asset index based on data from the entire sample on separate scores prepared for rural and urban households, and combined to produce a single asset index for all households and ranked into three (lowest, middle, and highest).

The difficulty of getting health services was assessed by the question “living closer to health facility” and the responses were categorized as “yes” or “no”. Media exposure was assessed based on whether people had access to read newsletters, listen to the radio, and watch TV. Accordingly, if they have access to all three media (newsletter, radio, and TV) at least once a week, we categorized them as “yes”, otherwise “no”.

### Data processing and analysis

The data were extracted, cleaned, re-coded, and analyzed using STATA version 17. The data were weighted using sampling weight during the statistical analysis to adjust for unequal probability of selection due to the sampling design used in DHS data. Tables and narrations were used to present the descriptive statistics. Since the DHS data are hierarchical (individual were nested within communities), a two-level binary logistic regression model was fitted to estimate the effect of both individual and community-level variables on PNC services uptake [32].

In this multilevel analysis, we fitted four models; i) Model 0: an empty (null) model without any explanatory variables, ii) Model 1: a model with individual-level variables, iii) Model 2: a model with community-level variables, and iv) Model 3: a model with both individual and community -level variables.

In the survey, the individual and household level data were nested under the community level data, for model comparison, Intra-class Correlation Coefficient and deviance (− 2* log likelihood ratio) were used. Accordingly, a model with lowest deviance was chosen. The variation between clusters was assessed by computing Intra-lass Correlation Coefficient (ICC) [33]. The ICC is the proportion of variance explained by the grouping structure in the population which: ICC=; Where, the standard logit distribution has a variance of,, indicates the cluster variance. The ICC greater than 5% is eligible for multilevel analysis and in our analysis, the ICC was 22.5%.

A mixed effect multilevel binary logistic regression analysis was done. A low deviance value was used to estimate the model goodness of fit by comparing the full model with the preceding three models. Finally, a *p*-value of less than 0.05 and an adjusted odds ratio (AOR) with 95% confidence interval (CI) were used to declare statistically significant factors associated with PNC services uptake among deviant women.

## Results

### Descriptions of the deviant women

Table 1 presents the descriptive statistics of deviant women in Ethiopia. A total of 2417 deviant women (women who had no education) were identified from the EDHS 2016 and included in this analysis. The mean age of deviant women was 29.6 ± 6.4 years, and 91.4% of the them were rural dwellers. Majority (66.1%) of the deviant women were in the lowest household wealth status and the mean family size was 6.3 ± 2.3. Furthermore, majority (59.8%) of the deviant women were Muslim religion followers.

**Table 1:** Socio-demographic and economic characteristics of deviant women in Ethiopia, EDHS 2016 (*n* = 2417)

Variables	Frequency (n)	Percentage (%)
Age (in years)		
15–24	506	20.9
25–34	1296	53.6
> =35	615	25.5
Religion		
Muslim	1446	59.8
Orthodox	609	25.2
Others^a^	362	15.0
Sex of head of household		
Male	1919	79.4
Female	498	20.6
Household wealth status		
Lowest	1597	66.1
Middle	346	14.3
Highest	474	19.6
Current marital status		
Married	2322	96.1
Unmarried	95	3.9
Respondent’s occupation		
No work	1563	64.7
Professional	191	7.9
Agricultural	495	20.5
Others^b^	168	6.9

^a^Others: Catholic, Traditional, Occupation ^b^Merchant, Self-employed, Daily labour

### Obstetric characteristics of the deviant women

Table 2 shows the obstetric characteristics of deviant women. Majority (49.4%) of them were grand multipara and 55.3% of them were had ANC visits for their recent pregnancy. There was a variation in place of delivery for their recent birth; almost three-fourth of the women gave their recent birth at home.

**Table 2:** Obstetric characteristics of deviant postnatal women in Ethiopia, EDHS 2016 (*n* = 2417)

Variables	Frequency (n)	Percentage (%)
Age at first birth (in years)		
< 18	958	39.6
18–24	1304	54.0
25+	155	6.4
Parity		
Primi-para	245	10.1
Multipara	979	40.5
Grand multipara	1193	49.4
ANC visit (s)		
No	1081	44.7
Yes	1336	55.3
Wanted their recent pregnancy		
Then	2001	82.8
Later	257	10.6
No more	159	6.6
Desire for more children		
Wants	1580	65.4
Wants no more	837	34.6
Place of delivery		
Home	1770	73.2
Health facility	647	26.8

*ANC* Antenatal care

### Community-level factors for PNC uptake among deviant women

Region, residence, exposure to mass media and distance to the health facility were the community level factors of PNC uptake among deviant women which are presented in Table 3. The analysis shows that only 10.4% of deviant postnatal women were had access to all media types (radio, newsletter, and television) more than once a week and 38.1% of the deviant women were living closer to health facility. A significant proportion (91.4%) of the deviant women were rural dwellers.

**Table 3:** Community-level factors for PNC uptake among deviant women in Ethiopia, EDHS 2016 (*n* = 2417)

Variables	Frequency (n)	Percentage (%)
Residence		
Urban	208	8.6
Rural	2209	91.4
Region		
Developed	1251	51.8
Emerging	1043	43.1
City administration	123	5.1
Exposure to mass media		
No	2166	89.6
Yes	251	10.4
Living closer to health facility		
No	1497	61.9
Yes	920	38.1

### PNC services uptake among deviant women

The overall PNC service uptake among deviant women was 5.8% (95% CI: 4.9–6.8). Of these who received PNC services, 5.7, 8.5, 34.0, 46.8% of the women were received PNC service by Doctors, Midwives, Health Extension Workers (HEWs) and Nurses, respectively, but the rest 5.0% were received PNC by others. Moreover, 14 (10.0%), 68 (48.2%), and 59 (41.8%) of the women received their PNC services within 24 hours, between 1 and 7th days, and after the 7th days, respectively.

### Individual and community level factors affecting PNC services uptake among deviant women

#### Random effect estimates

There was a significant variation of PNC services uptake among deviant women across the clusters. The intra-class correlation coefficient of PNC uptake among deviant women in the null model was 0.23 (95% CI: 0.02–0.81); meaning 23% of the variation in PNC services uptake among deviant women was due to the differences between clusters (between-cluster variation). The model fitness was check using the ICC across the four models and deviance. Accordingly, the null model (a model without independent variable) was scored a deviance of 1029, model 1 (a model with individual-level variables) was scored 979, model 2 (a model with community-level variables) was scored 1012, and model three (a model with both individual and community-level variables) was scored 970. Then a model with low deviance (model three) was chosen for the final analysis to identify the determinants of PNC uptake among the deviant women in Ethiopia.

#### Fixed effect estimates

The results presented in Table 4, after adjusting for individual and community level factors; women’s working in the agriculture, highest household wealth status, orthodox religion, residing in city administrations, and living closer to health facility were significantly increase the uptake of PNC services among women who had no education in Ethiopia.

**Table 4:** A mixed effect multilevel binary logistic regression analysis of individual and community-level factors associated with uptake of PNC services among deviant women in Ethiopia, EDHS 2016 (*n* = 2417)

Variables	PNC use		COR (95%CI)	Model 1 AOR (95% CI)	Model 2 AOR (95% CI)	Model 3 AOR (95% CI)
	Utilized n (%)	Non-utilized n (%)				
Age (in years)						
15–24	17 (3.36)	489 (96.64)	Ref.			
25–34	80 (6.17)	1216 (93.83)	1.91 (1.05–3.46)	1.45 (0.71–2.95)		1.41 (0.69–2.86)
35+	44 (7.15)	571 (92.85)	2.21 (1.16–4.21)	1.46 (0.63–3.38)		1.40 (0.60–3.23)
Marital status						
Married	134 (5.77)	2188 (94.23)	Ref.			
Not married	7 (7.37)	(88 (92.63)	1.32 (0.52–3.33)	1.38 (0.52–3.64)		1.36 (0.52–3.55)
Occupation						
No work	67 (4.29)	1496 (95.71)	Ref.			
Professional	13 (6.81)	178 (93.19)	1.70 (0.82–3.50)	1.17 (0.55–2.47)		1.22 (0.58–2.56)
Agricultural	48 (9.70)	447 (90.30)	2.63 (1.63–4.22)	2.05 (1.25–3.35)		2.15 (1.31–3.52) *
Others	13 (7.74)	155 (92.26)	1.95 (0.94–4.05)	1.54 (0.73–3.25)		1.54 (0.73–3.26)
Religion						
Muslim	56 (3.87)	1390 (96.13)	Ref.			
Orthodox	68 (11.17)	541 (88.83)	3.46 (2.08–5.75)	2.46 (1.44–4.23)		2.56 (1.42–4.57) *
Others	17 (4.70)	345 (95.30)	1.34 (0.67–2.68)	1.19 (0.59–2.42)		1.32 (0.65–2.70)
Household wealth index						
Lowest	66 (4.13)	1531 (95.87)	Ref.			
Middle	28 (8.09)	318 (91.91)	1.92 (1.12–3.28)	1.69 (0.98–2.92)		1.61 (0.93–2.80)
Highest	47 (9.92)	427 (90.08)	2.80 (1.72–4.56)	2.44 (1.48–4.05)		2.22 (1.25–3.91) *
Parity						
Primi-para	11 (4.49)	234 (95.51)	Ref.			
Multi-para	54 (5.52)	925 (94.48)	1.32 (0.62–2.81)	1.07 (0.45–2.50)		1.10 (0.47–2.57)
Grand multi-para	76 (6.37)	1117 (93.63)	1.78 (0.84–3.75)	1.34 (0.52–3.43)		1.37 (0.53–3.53)
Desire more child						
Wants	85 (5.38)	1495 (94.62)	Ref.			
Wants no more	56 (6.69)	781 (93.31)	1.24 (0.83–1.86)	0.88 (0.56–1.36)		0.89 (0.57–1.38)
Residence						
Urban	16 (7.69)	192 (92.31)	Ref.			
Rural	125 (5.66)	2084 (94.34)	0.63 (0.30–1.32)		0.89 (0.40–2.00)	1.30 (0.54–3.10)
Region						
Developed	87 (6.95)	1164 (93.05)	Ref.			
Emerging	41 (3.93)	1002 (96.07)	0.51 (0.30–0.87)		0.52 (0.31–0.88)	1.06 (0.59–1.90)
City administration	13 (10.57)	110 (89.43)	1.66 (0.65–4.22)		1.67 (0.63–4.37)	3.17 (1.15–8.71) *
Living closer to health facility						
Yes	76 (8.26)	844 (91.74)	Ref.			
No	65 (4.34)	1432 (95.66)	0.55 (0.37–0.83)		0.56 (0.36–0.84)	0.63 (0.41–0.96) *
Exposure to mass media						
No	123 (5.68)	2043 (94.32)	Ref.			
Yes	18 (7.17)	233 (92.83)	1.27 (0.69–2.31)		1.03 (0.55–1.94)	0.86 (0.44–1.65)

*AOR* Adjusted Odds Ratio, *COR* Crude Odds Ratio, *PNC* Postnatal care, Model 1: adjusted for individual-level factors, Model 2: adjusted for community-level factors, Model 3: adjusted for both individual and community-level factors (full model)

*Statistically significant at *p*-value < 0.05 in the full model

*Ref.* Reference category

Hence, the odds of PNC services uptake among deviant women who were working in the agriculture was 2.15 time higher than the odds of PNC services uptake among deviant women who do not have work (AOR = 2.15, 95% CI: 1.13–3.52). These Orthodox religion followers’ deviant women had 2.56 times higher odds of PNC services uptake than Muslim religion follower deviant women (AOR = 2.56, 95% CI: 1.42–4.57). The odds of PNC services uptake among women who are in the highest household wealth status was 2.22 times higher than these who are in the lowest household wealth status (AOR = 2.22, 95% CI: 1.25–3.91). Deviant women reside in the city administration had 3.17 times higher odds of PNC services uptake than women who resides in the developed regions (AOR: 3.17, 95% CI: 1.15–8.71). These deviant women living closer to health facility had 1.57 times higher odds of PNC services uptake than their counter parts (AOR: 1.57, 95% CI: 1.03–2.39).

## Discussion

The study investigated the individual and community level determinants that significantly increase the PNC services uptake among women with no education in Ethiopia. Mothers with no education are at a particularly high risk of low uptake of PNC services and are consequently a key target group for intervention. Identifying the positive determinants with PD behavior might be used to orient high impact interventions to improve the health of women and newborns since significant proportion of the women and newborns are not getting services in Ethiopia. In that regard, the government should increase the community based behavioral change interventions, strengthen the existing user free services and community-based health extension program at household level to favor the disadvantaged groups.

Our study showed that 5.8% [95%CI: 4.9–6.8] of the deviant women (women who had no education) were utilized PNC services in Ethiopia. This finding is low compared to few studies previously conducted in Ethiopia in which PNC services uptake after birth in prior studies range from 8 to 66.83% [10, 13, 14, 16, 22, 34]. The possible explanation for this variation might be the difference in study participants in which our study was conducted for a specific target group (women with no education). Moreover, the finding is also low compared to other studies conducted in India 26% [35], Zambia 28% [36], Nigeria 29% [26], Kenya 47% [17], and Bangladesh 73.5% [37]. This discrepancy might be due to the differences in study settings, socio-economic, cultural practices after birth, and also manly due to the differences in study participants; for instant, these studies included both women with education and no education, whereas in our analysis, only women without education. Mothers who did not receive a formal education may be unaware of the benefits of PNC services. Furthermore, women without formal education are less likely to engage in paid work and are more financially dependent on others, as a result, they may be unable to obtain PNC services. This implies education is the major predictor of health services use according to the Anderson’s model [29]. So, increasing women’s awareness on maternal health services using the existing community-based platforms is very crucial to bring a change on services utilization.

We identified that the employment status of women, religion, household wealth status, region and distance to a health facility were associated with uptake of PNC services among deviant women in Ethiopia, and in concert with prior studies using risk factor analysis [14, 16, 20, 21, 23, 34, 38–40]. The deviant women in the highest household wealth status were had higher uptake of PNC services. These findings are supported with that of previous studies conducted in Ethiopia [14, 38], India [39, 40] and Nigeria [23, 26] suggesting that being in a rich household is significantly increase the PNC service uptake. Women who could not afford to pay for direct and indirect medical costs still had difficulty accessing health facilities even though in most areas PNC services are provided for free [38]. The primary concern of poor households is meeting their basic needs and they may lack financial resources to cover the healthcare expenses [41, 42]. This implies that the government should establish interventions, initiatives, and expand existing community-based programs aimed at empowering women to generate their own income and be economically self-sufficient to get better access of health services. Moreover, the health extension program in Ethiopia that focus on household level and provision of user free services should be strengthen and reach at the grassroot level to increase the access to maternal and child health services.

In our study, Orthodox religion followers’ deviant women had higher odds of PNC services uptake than Muslim religion followers. Other studies conducted in Ethiopia also showed that mothers who followed the Ethiopian Orthodox religion use PNC services more than mothers belonging to other religions [38, 43]. This is highly likely due to differences in religious practices and beliefs. For instance, Muslim women are restricted from leaving their houses for 45 days after birth, whereas Christian women are permitted to leave their homes 10–15 days following childbirth [44]. In that regard, the government should aval the PNC services at household level within 7 days for these who give their birth at home that can solve the religious practices and beliefs and also transportation difficulties in accessing health care.

In our study, women who worked in agriculture were had better uptake of PNC services compared with those who do not have work. In contrast, a study conducted in Bench-Maji zone, Southern Ethiopia [20] showed that those mothers who were farmers were less likely to use PNC services in comparison with housewives. This discrepancy could be due to the difference in study participants and the socio-cultural practices specifically in the southern Ethiopia.

The deviant women reside in the city administration had higher PNC service uptake than women who resides in the developed regions. Women who reside in the city had more access to information, education and communication (IEC) services [20]. In addition, the health care providers and also the maternal health services might be more concentrated in the city compared to others and women who reside in the city may be more aware of health promotion initiatives, making it easier for them to access PNC services [45]. This implies that the government should put an effort to improve infrastructures, such as electricity, transportation, water, and sanitation as well as expand health facilities in order to improve the accessibility and quality of health facilities in order to enhance the uptake of PNC services in the peripheral regions of health facilities.

Previous studies have found that distance from the health institution remains a major concern and utilization of health services is strongly associated with access to health services [46–49]. In this study deviant woman living closer to health facility had higher PNC services uptake than their counterparts. Similarly, studies conducted in Africa and other developing countries have revealed that physical proximity [50, 51] and geographical distance [52] have a significant impact on the use of maternal health services. This implies that the Ethiopian government should provide maternity care close to the community by strengthening the maternity home initiatives in the primary health care units for these hard-to-reach areas and bringing the PNC services at household level.

### Strength and limitations of the study

This study is the first study that identify the positive determinants of PNC uptake among a specific disadvantaged group of population in Ethiopia using the positive deviance approach to orient high impact interventions. It was based on the weighted nationally representative data with a large sample size. Besides, the multilevel analysis was used to accommodate the hierarchical nature of the DHS data to get reliable standard error and estimates.

However, the study finding is interpreted in light of limitations. As with other cross-sectional studies, the temporal relationship cannot be established. Also, since data were collected from self-report from respondents there may be a possibility of recall bias.

## Conclusions

Only one in twenty deviant women (women who have no education) were received PNC services within 2 months after giving birth, which is extremely far behind the national target of PNC services utilization in Ethiopia. The positive deviance approach provides evidences for health policy makers and program implementers to identify factors facilitating improved health behavior, and ultimately better maternal and child health outcome in specific target population, despite an acknowledged adverse risk profile. Accordingly, deviant women (women who have no education) and working in the agriculture, being in the highest household wealth status, following orthodox religion, residing in city administrations, and living closer to the health facility were had a batter PNC uptake. Thus, the finding can be used to orient high impact maternal health services interventions and policy to improve the health status of these disadvantaged group of populations to meet the universal health coverage objectives. As a result, the government should improve the behavioral awareness creation on maternal health services using the existing community-based platform (community-based health extension program) and also empowering women through strengthening community-based women development army, increasing the access to maternal -health services through home based and user free services provision for these hard-to-reach areas.

## Data Availability

Data for this study were sourced from Demographic and Health surveys (DHS). The database was available at official website of DHS is at https://dhsprogram.com.

## Abbreviations

AOR: Adjusted Odds Ratio
CI: Confidence Intervals
COR: Crude Odds Ratio
DHS: Demographic and Health Survey
HEW: Health Extension Worker
ICC: Intra-Cluster Correlation
OR: Odds Ratio
PD: Positive Deviance
PNC: Postnatal Care
WHO: World Health Organization

## References

[1] World Health Organization. WHO technical consultation on postpartum and postnatal care. Geneva: World Health Organization; 2010. 26269861

[2] World Health Organization. Postpartum care of the mother and newborn: a practical guide: report of a technical working group. Geneva: World Health Organization; 1998. https://apps.who.int/iris/handle/10665/66439. 10655832

[3] Lawn JE, Kerber K, Enweronu-Laryea C, Cousens S (2010). 3.6 million neonatal deaths—what is progressing and what is not?. Seminars in perinatology.

[4] Ritchie MRaH (2020). Maternal mortality report our world in data.

[5] World Bank (2020). WHO Latest child mortality estimates.

[6] WHO (2020). WHO Newborn Mortality report..

[7] Blencowe H, Cousens S, Oestergaard MZ, Chou D, Moller A-B, Narwal R, Adler A, Garcia CV, Rohde S, Say L (2012). National, regional, and worldwide estimates of preterm birth rates in the year 2010 with time trends since 1990 for selected countries: a systematic analysis and implications. Lancet.

[8] Mekonnen W, Hailemariam D, Gebremariam A. Causes of maternal death in Ethiopia between 1990 and 2016: systematic review with meta-analysis. Ethiop J Health Dev. 2018;32(4):225–42.

[9] CSA (2016). Central statistical agency (CSA)[Ethiopia] and ICF.

[10] Tiruneh GT, Worku A, Berhane Y, Betemariam W, Demissie M (2020). Determinants of postnatal care utilization in Ethiopia: a multilevel analysis. BMC Pregnancy Childbirth.

[11] World Health Organization. WHO recommendations on postnatal care of the mother and newborn. Geneva: World Health Organization; 2014. https://apps.who.int/iris/handle/10665/97603. 24624481

[12] Tesfau YB, Kahsay AB, Gebrehiwot TG, Medhanyie AA, Godefay H (2020). Postnatal home visits by health extension workers in rural areas of Ethiopia: a cross-sectional study design. BMC Pregnancy and Childbirth.

[13] Regassa N. Antenatal and postnatal care service utilization in southern Ethiopia: a population-based study. Afr Health Sci. 2011;11(3):390. PMC3260999 22275929

[14] Berhe A, Bayray A, Berhe Y, Teklu A, Desta A, Araya T, Zielinski R, Roosevelt L (2019). Determinants of postnatal care utilization in Tigray, northern Ethiopia: a community based cross-sectional study. PLoS One.

[15] Akibu M, Tsegaye W, Megersa T, Nurgi S (2018). Prevalence and determinants of complete postnatal care service utilization in northern Shoa, Ethiopia. J Pregnancy.

[16] Wudineh KG, Nigusie AA, Gesese SS, Tesu AA, Beyene FY (2018). Postnatal care service utilization and associated factors among women who gave birth in Debretabour town, north West Ethiopia: a community-based cross-sectional study. BMC Pregnancy Childbirth.

[17] Akunga D, Menya D, Kabue M (2014). Determinants of postnatal care use in Kenya. Afr Popul Stud.

[18] Tessema ZT, Yazachew L, Tesema GA, Teshale AB (2020). Determinants of postnatal care utilization in sub-Saharan Africa: a meta and multilevel analysis of data from 36 sub-Saharan countries. Ital J Pediatr.

[19] Khaki J (2019). Factors associated with the utilization of postnatal care services among Malawian women. Malawi Med J.

[20] Abota TL, TadeleAtenafu N (2018). Postnatal care utilization and associated factors among married women in Benchi-Maji zone, Southwest Ethiopia: a community based cross-sectional study. Ethiop J Health Sci.

[21] Abuka Abebo T, Jember Tesfaye D (2018). Postnatal care utilization and associated factors among women of reproductive age Group in Halaba Kulito Town, Southern Ethiopia. Archives Public Health.

[22] Belachew T, Taye A, Belachew T (2016). Postnatal care service utilization and associated factors among mothers in Lemo Woreda, Ethiopia. J Women’s Health Care.

[23] Somefun OD, Ibisomi L (2016). Determinants of postnatal care non-utilization among women in Nigeria. BMC Research Notes.

[24] Manote M, Gebremedhin T (2020). Determinants of postnatal care non-utilization among women in Demba Gofa rural district, southern Ethiopia: a community-based unmatched case-control study. BMC Pregnancy Childbirth.

[25] Lwelamira J, Safari J, Stephen A (2015). Utilization of maternal postnatal care services among women in selected villages of Bahi District, Tanzania. Curr Res J Soc Sci.

[26] Dahiru T, Oche OM (2015). Determinants of antenatal care, institutional delivery and postnatal care services utilization in Nigeria. Pan African Med J.

[27] Angore BN, Tufa EG, Bisetegen FS (2018). Determinants of postnatal care utilization in urban community among women in Debre Birhan town, northern Shewa, Ethiopia. J Health Popul Nutr.

[28] Central Statistical Agency (CSA) [Ethiopia] and ICF (2016). Ethiopia Demographic and Health Survey 2016.

[29] Anderson JG (1973). Health services utilization: framework and review. Health Serv Res.

[30] Andersen RM. Revisiting the behavioral model and access to medical care: does it matter? J Health Soc Behav. 1995 ;36(1):1–0. 7738325

[31] Sisay MM, Geremew TT, Demlie YW, Alem AT, Beyene DK, Melak MF, Gelaye KA, Ayele TA, Andargie AA (2019). Spatial patterns and determinants of postnatal care use in Ethiopia: findings from the 2016 demographic and health survey. BMJ Open.

[32] Hox JJ, Moerbeek M, Van de Schoot R. Multilevel analysis: Techniques and applications. 3rd ed. New York: Routledge; 2010.

[33] Shimelis ND, Asticcioli S, Baraldo M, Tirillini B, Lulekal E, Murgia V (2012). Researching accessible and affordable treatment for common dermatological problems in developing countries. An Ethiopian experience. Int J Dermatol.

[34] Limenih MA, Endale ZM, Dachew BA (2016). Postnatal care service utilization and associated factors among women who gave birth in the last 12 months prior to the study in Debre Markos town, northwestern Ethiopia: a community-based cross-sectional study. Int J Reprod Med.

[35] Singh R, Neogi SB, Hazra A, Irani L, Ruducha J, Ahmad D, Kumar S, Mann N, Mavalankar D (2019). Utilization of maternal health services and its determinants: a cross-sectional study among women in rural Uttar Pradesh, India. J Health Popul Nutr.

[36] Jacobs C, Moshabela M, Maswenyeho S, Lambo N, Michelo C (2017). Predictors of antenatal care, skilled birth attendance, and postnatal care utilization among the remote and poorest rural communities of Zambia: a multilevel analysis. Front Public Health.

[37] Nayan SK, Begum N, Abid MR, Rahman S, Rajib AK, Farzana N, Nahar ML (2017). Utilization of postnatal care services among the rural women in Bangladesh. Northern Int Med Coll J.

[38] Mehari K, Wencheko E (2013). Factors affecting maternal health care services utilization in rural Ethiopia: a study based on the 2011 EDHS data. Ethiop J Health Dev.

[39] Yadav AK, Sahni B, Jena PK, Kumar D, Bala K (2020). Trends, differentials, and social determinants of maternal health care services utilization in rural India: an analysis from pooled data. Women’s Health Reports.

[40] Yadav AK, Jena PK, Sahni B, Mukhopadhyay D (2021). Comparative study on maternal healthcare services utilisation in selected empowered action group states of India. Health Social Care Community.

[41] Amin R, Shah NM, Becker S (2010). Socioeconomic factors differentiating maternal and child health-seeking behavior in rural Bangladesh: a cross-sectional analysis. Int J Equity Health.

[42] Singh L, Rai RK, Singh PK (2012). Assessing the utilization of maternal and child health care among married adolescent women: evidence from India. J Biosoc Sci.

[43] Mekonnen Y, Asnaketch M. Utilization of Maternal Health Care Services in Ethiopia: Ethiopian Health and Nutrition Research Institute. Calverton: ORC Macro; 2002.

[44] Zeleke LB, Wondie AT, Tibebu MA, Alemu AA, Tessema MT, Shita NG, Khajehei M (2021). Postnatal care service utilization and its determinants in east Gojjam zone, Northwest Ethiopia: a mixed-method study. PLoS One.

[45] Mekonnen Y, Mekonnen A. Utilization of Maternal Health Care Services in Ethiopia: Ethiopian Health and Nutrition Research Institute. Calverton: ORC Macro; 2002.

[46] Titaley CR, Hunter CL, Heywood P, Dibley MJ (2010). Why don't some women attend antenatal and postnatal care services?: a qualitative study of community members’ perspectives in Garut, Sukabumi and Ciamis districts of West Java Province, Indonesia. BMC Pregnancy Childbirth.

[47] Rahman M (2009). The determinants of use of postnatal care services for mothers: does differential exists between urban and rural areas in Bangladesh. Internet J Epidemiol.

[48] Warren C. Care seeking for maternal health: challenges remain for poor women. Ethiop J Health Dev. 2010;24(1 Special issue):100–4.

[49] Warren C. Care of the newborn: community perceptions and health seeking behavior. Ethiop J Health Dev. 2010;24(Special Issue 1):110–4.

[50] Stock R (1983). Distance and the utilization of health facilities in rural Nigeria. Soc Sci Med.

[51] Tarekegn SM, Lieberman LS, Giedraitis V (2014). Determinants of maternal health service utilization in Ethiopia: analysis of the 2011 Ethiopian demographic and health survey. BMC Pregnancy Childbirth.

[52] Rahaman MM, Aziz K, Munshi M, Patwari Y, Rahman M (1982). A diarrhea clinic in rural Bangladesh: influence of distance, age, and sex on attendance and diarrheal mortality. Am J Public Health.

